# Mechanism of High-Level Daptomycin Resistance in *Corynebacterium striatum*

**DOI:** 10.1128/mSphereDirect.00371-18

**Published:** 2018-08-08

**Authors:** Nicholas K. Goldner, Christopher Bulow, Kevin Cho, Meghan Wallace, Fong-Fu Hsu, Gary J. Patti, Carey-Ann Burnham, Paul Schlesinger, Gautam Dantas

**Affiliations:** a Edison Family Center for Genome Sciences & Systems Biology, Washington University in St. Louis School of Medicine, St. Louis, Missouri, USA; b Department of Chemistry, Washington University in St. Louis, St. Louis, Missouri, USA; c Department of Medicine, Washington University School of Medicine, St. Louis, Missouri, USA; d Department of Pathology and Immunology, Division of Laboratory and Genomic Medicine, Washington University School of Medicine, St. Louis, Missouri, USA; e Division of Endocrinology, Metabolism & Lipid Research, Washington University School of Medicine, St. Louis, Missouri, USA; f Department of Pediatrics, Washington University School of Medicine, St. Louis, Missouri, USA; g Department of Cell Biology and Physiology, Washington University School of Medicine, Saint Louis, Missouri, USA; h Department of Biomedical Engineering, Washington University, St. Louis, Missouri, USA; i Department of Molecular Microbiology, Washington University School of Medicine, St. Louis, Missouri, USA; Antimicrobial Development Specialists, LLC; McMaster University; Harvard Medical School

**Keywords:** *Corynebacterium*, antimicrobial resistance, artificial liposomes, daptomycin, genomics, lipidomics, phosphatidylglycerol, surface plasmon resonance, transcriptomics

## Abstract

Daptomycin, a last-line-of-defense antibiotic for treating Gram-positive infections, is experiencing clinical failure against important infectious agents, including Corynebacterium striatum. The recent transition of daptomycin to generic status is projected to dramatically increase availability, use, and clinical failure. Here we confirm the genetic mechanism of high-level daptomycin resistance (HLDR; MIC = >256 µg/ml) in C. striatum, which evolved within a patient during daptomycin therapy, a phenotype recapitulated *in vitro*. In all 8 independent cases tested, loss-of-function mutations in phosphatidylglycerol synthase (*pgsA2*) were necessary and sufficient for high-level daptomycin resistance. Through lipidomic and biochemical analysis, we demonstrate that daptomycin’s activity is dependent on the membrane phosphatidylglycerol (PG) concentration. Until now, the verification of PG as the *in vivo* target of daptomycin has proven difficult since tested cell model systems were not viable without membrane PG. C. striatum becomes daptomycin resistant at a high level by removing PG from the membrane and changing the membrane composition to maintain viability. This work demonstrates that loss-of-function mutation in *pgsA2* and the loss of membrane PG are necessary and sufficient to produce high-level resistance to daptomycin in C. striatum.

**IMPORTANCE** Antimicrobial resistance threatens the efficacy of antimicrobial treatment options, including last-line-of-defense drugs. Understanding how this resistance develops can help direct antimicrobial stewardship efforts and is critical to designing the next generation of antimicrobial therapies. Here we determine how Corynebacterium striatum, a skin commensal and opportunistic pathogen, evolved high-level resistance to a drug of last resort, daptomycin. Through a single mutation, this pathogen was able to remove the daptomycin’s target, phosphatidylglycerol (PG), from the membrane and evade daptomycin’s bactericidal activity. We found that additional compensatory changes were not necessary to support the removal of PG and replacement with phosphatidylinositol (PI). The ease with which C. striatum evolved high-level resistance is cause for alarm and highlights the importance of screening new antimicrobials against a wide range of clinical pathogens which may harbor unique capacities for resistance evolution.

## Abstract (precis)

Antimicrobial resistance threatens the efficacy of antimicrobial treatment options, including last-line-of-defense drugs. Understanding how this resistance develops can help direct antimicrobial stewardship efforts and is critical to designing the next generation of antimicrobial therapies. Here we determine how Corynebacterium striatum, a skin commensal and opportunistic pathogen, evolved high-level resistance to a drug of last resort, daptomycin. Through a single mutation, this pathogen was able to remove the daptomycin’s target, phosphatidylglycerol (PG), from the membrane and evade daptomycin’s bactericidal activity. We found that additional compensatory changes were not necessary to support the removal of PG and replacement with phosphatidylinositol (PI). The ease with which C. striatum evolved high-level resistance is cause for alarm and highlights the importance of screening new antimicrobials against a wide range of clinical pathogens which may harbor unique capacities for resistance evolution.

## INTRODUCTION

Current trends in increasing antibiotic resistance and decreasing drug development require urgent mitigation (1–3). Antibiotic-resistant infections claim over 700,000 lives globally each year, and this annual toll is predicted to swell to 10 million deaths a year by 2050 without significant intervention. A growing number of bacterial infections are already resistant to virtually all first-line antibiotics (2, 4). Physicians are forced to use “last-resort,” broad-spectrum antibiotics more frequently, and resistance to even these carefully safeguarded drugs has emerged (5, 6). Daptomycin is one such last-resort nonlytic (7) lipopeptide antibiotic and is effective against both stationary-phase and log-phase Gram-positive bacterial pathogens (8), including Staphylococcus aureus, Enterococcus faecium, and Corynebacterium striatum (9–11). Daptomycin integrates Ca^2+^ dependently into the bacterial cell membrane, causing membrane dysfunction that leads to K^+^, Mg^2+^, and ATP leakage and cell death (12, 13). Very low levels of resistance were observed during the early phase of daptomycin’s clinical use. Regrettably, recent clinical reports of treatment failures have emerged, with target pathogens exhibiting >2,000-fold increases in daptomycin resistance (DR) (14–16), often over short time scales (hours to a few days of treatment), which are beginning to challenge daptomycin’s efficacy. These failures are expected to expand, as daptomycin use is predicted to increase dramatically because its recent transition to generic status (17) will increase its availability for clinical use.

C. striatum is an emerging opportunistic pathogen that colonizes the skin much like S. aureus and has the ability to rapidly transition from susceptible to resistant to the critical antibiotic daptomycin. This work establishes a genetic, transcriptomic, lipidomic, and biochemical understanding of how C. striatum rapidly evolves high-level daptomycin resistance (HLDR) which is mechanistically distinct from that seen with S. aureus and *Streptomyces* spp. In S. aureus, low-level, stepwise accumulations in resistance phenotypes are responsible for low (MIC, 2 to 4 µg/ml) and intermediate (MIC, 4 to 8 µg/ml) daptomycin resistance. The majority of these observations come from pathogenic S. aureus, which was the first approved therapeutic target for daptomycin (18). Accumulation of multiple single nucleotide polymorphisms (SNPs) in the *yycFGHI* operon in S. aureus has resulted in 2-to-6-fold increases in daptomycin resistance through cell wall thickening and alteration of membrane charge (19–24). Increases in levels of positively charged membrane phospholipids, which reduce the affinity of the Ca2^+^-conjugated daptomycin for the surface membrane, increased the MIC. Additionally, mutations that alter lipid translocation and decrease membrane fluidity and thickening of the cell wall have led to low-level (3-to-6-fold) increases in daptomycin resistance (21, 25). Mutations associated with the physiological changes in pathogenic S. aureus described above have all led to small (2-to-6-fold) stepwise increases in resistance over long periods of time (weeks of treatment) and to loss of resistance to daptomycin when the strain is no longer under daptomycin’s selection pressure (26). In contrast, some environmental *Streptomyces* species have been shown to inactivate daptomycin enzymatically (27, 28); clinical isolates have not used this mechanism to date.

The first report of higher levels (~20-fold over the wild-type [WT] strain) of daptomycin resistance came from laboratory adaptive evolution experiments performed with the nonpathogenic soil bacterium Bacillus subtilis (29). Daptomycin-resistant B. subtilis was found to harbor SNPs in 44 genes, including predicted reduction/loss-of-function mutations in phosphatidylglycerol (PG) synthase A (*pgsA*), an essential enzyme for PG synthesis (29, 30). Characterization of the lipid membrane revealed a reduction in PG content from 30% in the wild-type strain to 10% in the resistant mutant. Consistent with the lack of complete ablation of PG in the membrane, attempts to knock out *pgsA* alone genetically were not successful due to the presumed essentiality of PG in B. subtilis (29). Nevertheless, studies of daptomycin’s target to date corroborate *in vivo* the importance of PG in daptomycin activity (29–31). Indeed, a recent comparative genomic and lipidomic study of S. aureus, C. striatum, and Enterococcus faecalis indicated that mutations in PG synthase and the subsequent lack of PG synthesis confer daptomycin resistance (31).

Over the past few years, there has been a steady increase in reports of even higher levels of daptomycin resistance (≥4,000-fold increases in resistance) in a number of clinical pathogens, including viridians group streptococci (15), Enterococcus faecium (11), and C. striatum (14). This high-level daptomycin resistance (HLDR)—defined here as a MIC of ≥256 µg/ml daptomycin—was first observed in C. striatum, in a patient with native valve endocarditis in 2012 (32). In 2014, a clinical laboratory reported *in vivo* evolution of HLDR C. striatum in a patient with an infected left ventricular assist device, during 17 days of daptomycin therapy (14). Evolution of HLDR was recapitulated *in vitro* in 100% of tested C. striatum isolates (*n* = 50) after 24 h of daptomycin exposure (33). C. striatum is a Gram-positive bacterium which typically resides as a commensal organism on the skin (34). However, it has become a growing threat to hospital systems and patients as an opportunistic pathogen. Indeed, C. striatum has been associated with a plethora of infection types over the past 20 years, including bacteremia, endocarditis, urinary tract, wound, respiratory, central line, medical device, and hardware infections (14, 35–41, 72). Here we used a combination of comparative genomics, transcriptomics, lipidomics, electron microscopy, and biochemical lipid and liposome characterization to elucidate the mechanism of HLDR evolved in C. striatum both within patients and *in vitro*. We demonstrate that loss-of-function mutations in *pgsA2* resulted in reductions in the levels of PG, the primary target of this last-resort drug, from ~45% to <1% in the membrane, resulting in HLDR. Our work demonstrates that this is an important consideration for designing future antibiotics that target the membrane since bacteria can manipulate lipid composition to effectively evade lipid targeted lipopeptides.

## RESULTS

**FIG 1:**
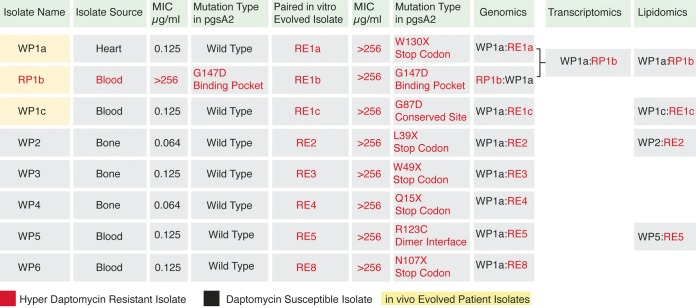
Available and tested C. striatum isolates. Isolate naming convention: W, WT; R, resistant; P, isolated from a patient; E, evolved from a patient isolate in culture under conditions of daptomycin selection; numbers, different isolate sources; a, b, and c suffixes, isolates collected from the same patient at different time points. All isolates’ genomes were sequenced. WP1a was used as the reference genome. All other genomes were mapped to that genome, and SNPs found in the resistant isolate and not the susceptible isolate from each pair were analyzed further. Transcriptomic analysis was performed on WPIa and RP1b. Lipidomics analysis was performed on the WT strain and the resistant matched pairs of: WP1a:RP1b, WP1c:RE1c, WP2:RE2, and WP5:RE5.

Analysis was performed on an evolved inpatient HLDR isolate (RP1b) and seven evolved *in vitro* HLDR isolates. These strains were banked clinical C. striatum isolates (Fig. 1). Paired susceptible strains obtained prior to HLDR evolution were used as controls. A total of 17 days elapsed between collection of the inpatient susceptible strain and HLDR isolate collection, and 24 h elapsed between collection of the *in vitro* susceptible strain and HLDR isolate collection.

### PG synthase mutations in HLDR C. striatum strains.

**FIG 2:**
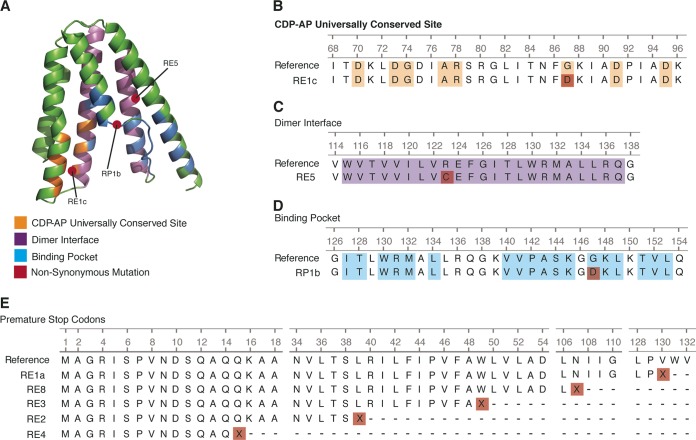
All HLDR isolates have predicted nonfunctional mutations in *pgsA2*. (A) Structure of PG synthase monomer, with mutations in conserved sites overlaid. (B) Mutation in CDP alcohol phosphatidyl (CDP-AP) transferase active site conserved across species. (C) Mutation in the dimer interface domain. (D) Mutation in substrate binding pocket. (E) Premature stop mutations predicted to produce truncated products.

All cases of evolved high-level daptomycin resistance (HLDR) in C. striatum that were tested (Fig. 1 and 2) had a predicted loss-of-function mutation in PG synthase. We performed whole-genome sequencing of eight pairs of *in vivo* and *in vitro* evolved HLDR C. striatum isolates (*n* = 16) and found *pgsA2* to be the only gene mutated consistently in all HLDR mutants. The affected gene encodes PG synthase, responsible for converting cytidine diphosphate diacylglycerol (CDP-DAG) to PG (Fig. 2A). The mutations observed included coding changes at universally conserved sites (Fig. 2B), the dimer interface (Fig. 2C), the active site (Fig. 2D), and those leading to premature stop codons (Fig. 2E). Each of these mutations is predicted by snpEFF (42, 43) and PHYREII (44, 45) homology modeling to result in loss of PG synthase activity.

We found no additional SNPs in the C. striatum genomes predicted to alter cellular biosynthetic processes in potential compensation for PG synthase loss of function. In B. subtilis, loss of just *pgsA2* (PG synthase) was lethal and compensatory mutations were necessary for cell survival (29), leading us to consider whether additional mutations may also be required for HLDR in C. striatum. A total of 8 additional nonsynonymous SNPs in biosynthetic pathways were detected in the *in vivo* evolved isolate (see Fig. S1 in the supplemental material). Aside from the *pgsA2* mutation, no SNPs in biosynthetic pathways were detected in the *in vitro* evolved HLDR isolates. This is consistent with the longer time between susceptible and resistant isolate collection *in vivo* (17 days) versus *in vitro* (24 h). The remaining SNPs in genes not related to biosynthesis did not cluster in similar pathways (Data Set S1). No consistent genetic change besides the loss-of-function mutation in *pgsA* is predicted to result in compensatory changes that would contribute to membrane viability or HLDR in C. striatum.

10.1128/mSphereDirect.00371-18.1 FIG S1  Nonsynonymous mutations in biosynthetic pathways. SNPs were clustered by functional category (using gene ontology). We found that *pgsA2* was the only gene related to cellular biosynthetic function that was mutated, with the exception of 8 additional mutations observed only in the *in vivo* evolved isolate. These mutations are consistent with the longer evolutionary time (17 days) experienced by the isolate between the susceptible and resistant states, in contrast to the *in vitro* evolution of only 24 h. Download FIG S1, PDF file, 0.3 MB. Copyright © 2018 Goldner et al. 2018 Goldner et al. This content is distributed under the terms of the Creative Commons Attribution 4.0 International license.

10.1128/mSphereDirect.00312-18.5 DATA SET S1 Raw data. Download DATA SET S1, XLSX file, 0.1 MB. Copyright © 2018 Goldner et al. 2018 Goldner et al. This content is distributed under the terms of the Creative Commons Attribution 4.0 International license.

All of the parent daptomycin-susceptible C. striatum isolates were derived from different patients, and the *in vivo* and *in vitro* HLDR phenotypes were evolved independently (Fig. 1). This breadth of evolution events in C. striatum isolates obtained from infected patients provides a robust, clinically relevant cohort for assessing mutations necessary to daptomycin resistance. Accordingly, comparative genomics indicated that loss-of-function SNPs in *pgsA2* encoding PG synthase represent the only genomic change necessary for HLDR and that no additional mutations are required to maintain resistant cell viability (Fig. 2). In a recent report of evolved xenobiotic resistance in *Corynebacterineae* (the suborder which includes *Corynebacterium*), minimal genetic mutations were observed between susceptible and resistant pairs, but large-scale transcriptomic changes were found to explain the change in phenotype (31, 46). Accordingly, we tested whether whole-cell transcriptional changes were potentially responsible for compensating for the loss of PG synthase function and stabilization of the membrane in HLDR C. striatum.

### Minimal transcriptional changes in HLDR C. striatum*.*

**FIG 3:**
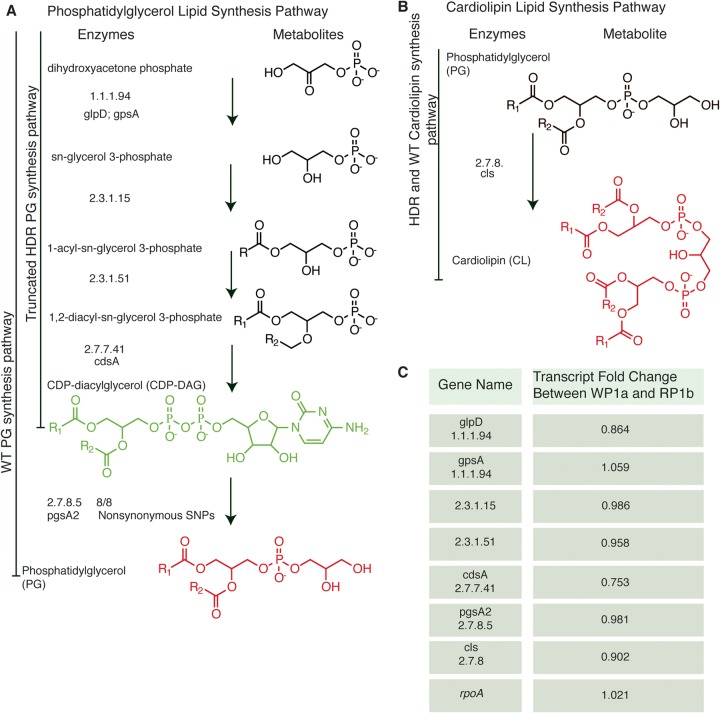
Lipid metabolism pathway of phosphatidylglycerol and cardiolipin, observed SNPs, and relative abundance changes of key lipids and transcripts between WT and HLDR isolates. (A and B) The PG (A) and CL (B) lipid synthesis pathways were constructed with KEGG. A total of 8 WT and HLDR isolate paired genomes were compared and nonsynonymous single nucleotide polymorphisms identified. The names and structures of the metabolites are on the left, with key lipids colored in red and green. R1 represents the 16:0 carbon chain, and R2 represents the 18:1 carbon chain. The enzyme nomenclature for each enzymatic step and the corresponding genes are located next to the appropriate synthesis arrow. SNP mutations for each enzyme/gene unit are indicated at the far right with the number of mutations among the 8 HLDR isolates, unless no mutations were present in any of the isolates. Except for *pgsA2*, none of the lipid synthesis genes for PG have SNPs. On the left side, black bars indicate the functional completeness of the PG synthesis pathway. The WT strain proceeds through the entire synthesis pathway producing PG, while the HLDR strain ends at the production of CDP-DAG. The green coloring of the lipid CDP-DAG indicates a 543-fold to 5,946-fold buildup of that metabolite in the HLDR isolates compared to the WT. The red coloring of PG and cardiolipin indicates a reduction of that metabolite in the HLDR isolates compared to the WT with a 369- to 1,990-fold reduction in PG. (C) Expression levels of genes involved in lipid synthesis pathways were not significantly altered in HLDR isolates (*P* > 0.05). Expression levels of the housekeeping genes *rpoA* and *gyrA* were also not significantly altered (*P* > 0.05).

No significant transcriptional changes were detected in biosynthetically linked genes in clinically evolved HLDR in C. striatum (Fig. 3C and Data Set S1; see also Fig. S2B). We compared the transcriptomes of the WP1a strain (index; daptomycin susceptible) and the RP1b strain (HLDR evolved in the patient) grown in cation-adjusted Mueller-Hinton broth (CAMHB) to the exponential phase in biological triplicate. Even the transcriptional changes of the largest magnitude did not exceed ±85%, representing a magnitude much smaller than that of the transcriptomic changes typically associated with phenotypic alteration (46). Expression changes in genes related to phospholipid biosynthesis were small in magnitude (less than 25%) and not statistically significant (Fig. 3C; see also Fig. S2). Furthermore, most transcriptomic changes observed occurred in transposase genes and hypothetical proteins of viral origin. The most notable biosynthetically linked change was the LGFP repeat protein transcript detected at levels 1.302-fold (~30%) greater in the HLDR strain. *pgsA2* expression levels changed by only 0.981-fold (not statistically significant) in the HLDR strain, further indicating that the HLDR phenotypic consequence of the predicted PG synthase loss-of-function binding pocket mutation in the RP1b strain was due to loss of activity rather than loss of expression. Lack of transcriptional alterations prompted us to interrogate the membrane composition of daptomycin-susceptible and HLDR C. striatum strains using a comparative lipidomics approach. We hypothesized that the HLDR phenotype resulted from the disruption of PG synthase activity, which effectively removed PG from the membrane (Fig. 3A).

10.1128/mSphereDirect.00371-18.2 FIG S2  Observed phosphatidylinositol SNPs and relative changes in the abundance of key lipids and transcripts between WT and HLDR isolates. The lipid synthesis pathways were constructed with KEGG. A total of 8 paired WT and HLDR isolate genomes were compared and nonsynonymous single nucleotide polymorphisms identified. The names and structures of the metabolites are indicated on the left, with key lipids colored in green. R1 represents the 16:0 carbon chain, and R2 represents the 18:1 carbon chain. The enzyme nomenclature for each enzymatic step and the corresponding genes are given next to the appropriate synthesis arrow. SNP mutations for each enzyme/gene unit are indicated at the far right with the number of mutations among the 8 HLDR isolates, unless no mutations were present in any of the isolates. On the left side, black bars indicate the functional completeness of the PI synthesis pathway. WT and HLDR proceed through the entire synthesis pathway producing PI. The green coloring of the PI lipid indicates a 0.65-fold to 4.25-fold buildup of that metabolite in the HLDR isolates compared to the WT strain. Fold change was calculated using (*b* − *a*)/*a*, where “*b*” is the largest value and “*a*” is the smallest (to maintain a positive number). Download FIG S2, PDF file, 0.3 MB. Copyright © 2018 Goldner et al. 2018 Goldner et al. This content is distributed under the terms of the Creative Commons Attribution 4.0 International license.

### Lipidomics reveals loss of phosphatidylglycerol in the membrane.

**FIG 4:**
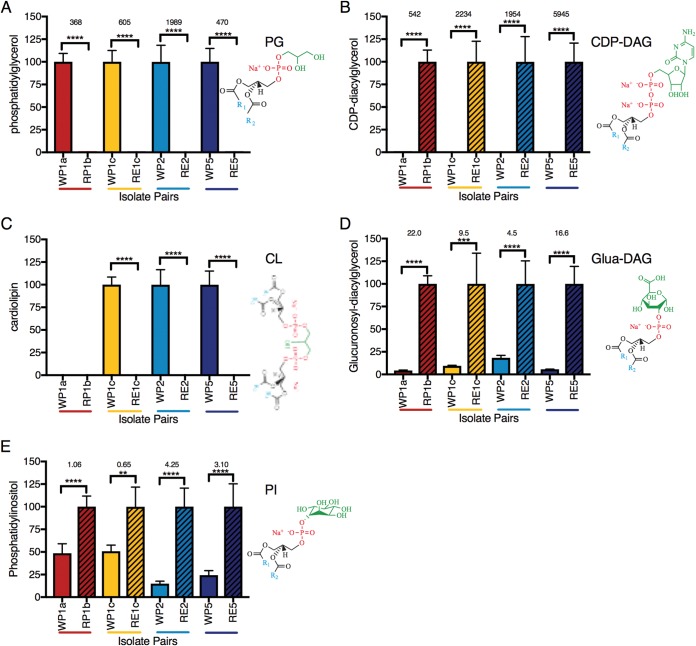
Lipidomic comparison of WT and resistant paired isolates across mutation types. (A to E) The *y* axis represents the relative abundance of the important phospholipid between the WT and HLDR isolates. WT-HLDR pairs are associated by color, with WT represented by solid block colors and HLDR represented by black-striped colors. The value corresponding to the lipid that is most abundant in the WT or HLDR isolate has been normalized to 100. Fold changes, where calculable, are listed above the WT and HLDR comparisons; fold change was calculated using (*b* − *a*)/*a*, where “*b*” is the largest value and “*a*” is the smallest, to maintain a positive number. The structures of the lipid are directly to the right of the graph, where R_1_ represents the 16:0 carbon chain and R_2_ represents the 18:1 carbon chain. Statistical analysis was performed with 1-way ANOVA, and the data in every column represent comparisons of the means (*, *P* ≤ 0.05; **, *P* ≤ 0.01; ***, *P* ≤ 0.001; ****, *P* ≤ 0.0001).

We found that loss of PG synthase function led to removal or at least a >360-fold reduction of membrane phosphatidylglycerol (PG) content in HLDR C. striatum isolates. Analysis by mass spectrometry (MS) of the whole-cell membrane lipid content of four pairs of daptomycin-susceptible and HLDR isolates, which represent each of the four types of predicted loss-of-function *pgsA2* mutations (Fig. 1, 2B to E, and 3A and B), reveals loss of PG. In each isolate pair, PG detection was 369-fold to 1,990-fold (*P* ≤ 0.0001) lower in the evolved HLDR isolates than in the daptomycin-susceptible ancestor (Fig. 4A), levels in the HLDR isolates which are indicative of complete removal of PG in the membrane, resulting in the HLDR phenotype. In addition to PG, cardiolipin (CL) (a derivative of PG) was the other lipid absent in the HLDR isolates (Fig. 4A and C). However, CL was also absent in the daptomycin-susceptible WP1a isolate, and on the basis of subsequent *in vitro* data, we posit that it was not the primary target of daptomycin. We also found no lipidomic evidence that the sn-1/sn-2 fatty acyl groups in PG are being modified to shield it from daptomycin binding as hypothesized in S. aureus isolates with low-level resistance (47–50). Conversion of PG to CL, a proposed mechanism of daptomycin resistance (51), also did not contribute to the C. striatum HLDR mechanism, since CL was absent in the HLDR strains (*P* ≤ 0.0001) (Fig. 4C). PG synthase converts CDP-DAG, a biosynthetic precursor of PG, into phosphatidyl glycerol (Fig. 3A). In the absence of PG synthase, we expected CDP-DAG either to be utilized in a secondary lipid synthesis pathway or to accumulate in the cell. In support of the latter hypothesis, we found that CDP-DAG levels were significantly (543-fold to 5,946-fold; *P* ≤ 0.0001) higher in HLDR isolates than in their wild-type (WT) counterparts (Fig. 3A and 4B). PG synthase in HLDR strains across mutation types was nonfunctional (Fig. 2B to E and 3A), and no transcriptional compensatory changes were being made in the PG biosynthesis pathway (Fig. 3; see also Fig. S2), enabling high-level CDP-DAG accumulation (Fig. 3A and 4).

**FIG 5:**
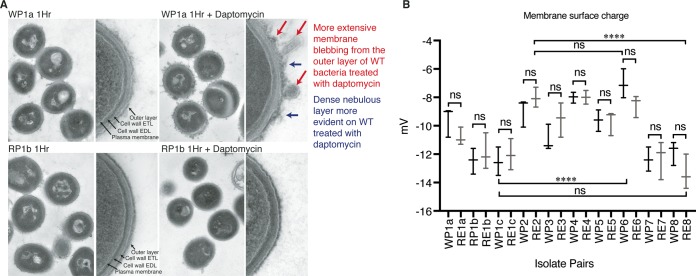
Comparison of HLDR and WT lipid membranes and surface charge. (A) WP1a and RP1b isolates were imaged with transmission electron microscopy after 1 h with or without the addition of 10 µg/ml daptomycin. WP1a without daptomycin and RP1b with and without daptomycin showed no membrane irregularities, while WP1a with daptomycin showed membrane blebbing and disruption. EDL, electron-dense layer; ETL, electron-transparent layer. (B) All available WT and HLDR isolate pairs were checked for surface membrane charge changes. Charges were not indicative of HLDR, and the differences in the ranges of surface charge between the most and least negative WT and HLDR isolates were not significant, while there was significant variability in charge for both the WT and HLDR isolates compared within MIC group. Statistical analysis was performed with 1-way ANOVA and paired-means analysis (ns, *P* > 0.05; *, *P* ≤ 0.05; **, *P* ≤ 0.01; ***, *P* ≤ 0.001; ****, *P* ≤ 0.0001).

**FIG 6:**
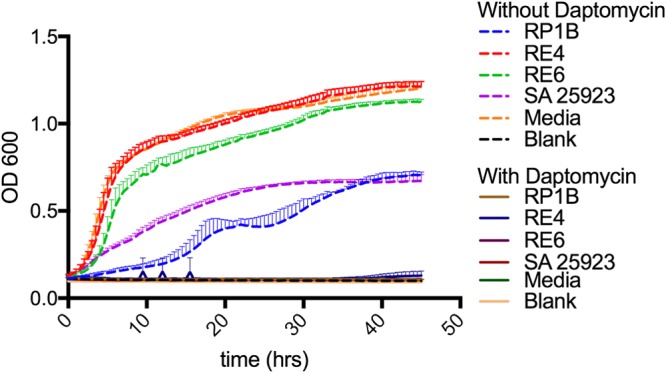
Daptomycin spent-medium growth curves. Growth curves of daptomycin-susceptible S. aureus ATCC 29213 grown in spent media are shown. Spent medium was obtained by growth of resistant isolates (RP1b, RE4, and RE6) or the control isolate (S. aureus [SA] 25923) and uninoculated media, with or without daptomycin (5 µg/ml), for 24 h. After filtration, in triplicate, susceptible S. aureus ATCC 29213 grew only in media that had never contained daptomycin. This indicates lack of daptomycin degradation by HLDR isolates. OD 600, optical density at 600 nm.

Until recently, determining the *in vivo* target of daptomycin had been challenging because removal of PG from the membrane of model Gram-positive bacteria without cell death was biologically untenable (16, 52). A recent study (31) corroborated that C. striatum appears to uniquely compensate for the complete removal of PG in its membrane by increasing the proportions of two other lipids in the membrane: phosphatidylinositol (PI) (Fig. 4E; see also Fig. S2A and B) and glucuronosyl diacylglycerol (Glua-DAG) (Fig. 4D). The level of PI was 1.6-fold to 5.3-fold higher (*P* ≤ 0.0001 and *P* ≤ 0.001) in the HLDR isolates than in the WT (Fig. 4E; see also Fig. S3A), and the level of Glua-DAG was 5.5-fold to 23.0-fold higher (*P* ≤ 0.0001 and *P* ≤ 0.01) in the HLDR isolates than in the WT (Fig. 4D). We demonstrated that the previously proposed mechanisms of daptomycin resistance, which include altering membrane fluidity, leaflet organization, and morphology, do not contribute to HLDR in C. striatum, as the lipid membrane composition changes in the HLDR isolates did not visibly alter the membrane (by transmission electron microscopy; Fig. 5A) or charge (by zeta potential; Fig. 5B) compared to their daptomycin-susceptible counterparts. Furthermore, daptomycin is very stable in solution and resistant to degradation. Additionally, WT C. striatum evolved resistance to daptomycin in monoculture, and it is therefore unlikely that C. striatum acquired new antibiotic resistance genes that could degrade daptomycin, especially given that HLDR developed in eight independent cases of evolution with the same genomic and phenotypic changes. Additionally, the results of spent-medium experiments performed with three HLDR C. striatum isolates and with one susceptible S. aureus isolate indicated that daptomycin was not degraded, as susceptible S. aureus was unable to grow on daptomycin-containing spent media but was able to grow in control spent media that did not contain daptomycin (Fig. 6). These data affirm that PG is the *in vivo* target of daptomycin and that loss of PG due to nonfunctional PG synthase is necessary and sufficient for the HLDR phenotype.

10.1128/mSphereDirect.00371-18.3 FIG S3  Structures of the key phospholipids. These lipids are critical to bacterial membrane composition. R_1_, 16:0 carbon chain; R_2_, 18:1 carbon chain. Black indicates the glycerol backbone, red indicates the phosphate group, and green represents the functional group. Download FIG S3, PDF file, 0.3 MB. Copyright © 2018 Goldner et al. 2018 Goldner et al. This content is distributed under the terms of the Creative Commons Attribution 4.0 International license.

### Surface plasmon resonance analysis indicates that PG is the preferred target of daptomycin.

**FIG 7:**
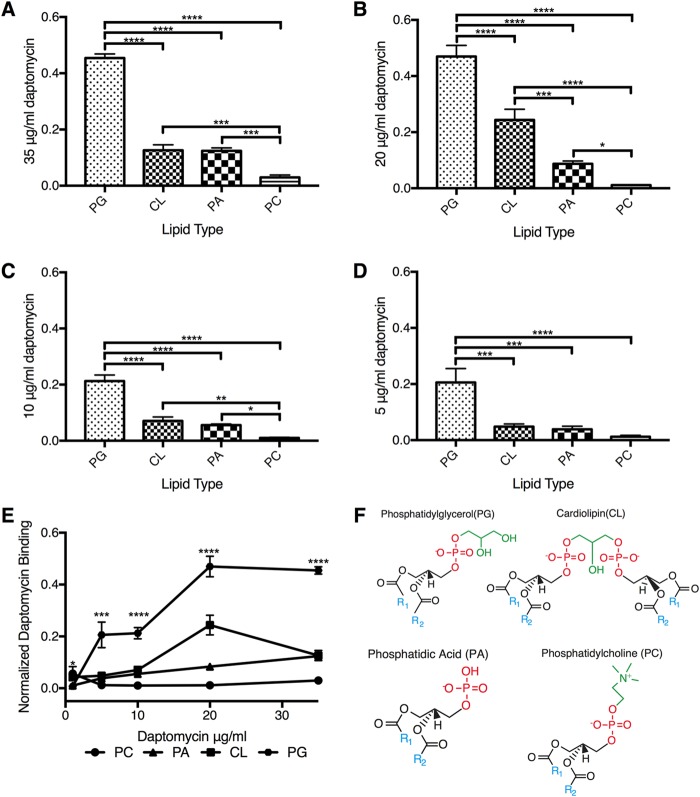
Daptomycin binding across concentrations and liposome content. (A to E) The *y* axis plots the normalized binding of daptomycin to liposomes at various concentration ratios. The *x* axis shows the types of liposomes tested, which include 1:1 equimolar ratios of PG, PA, or CL to PC and a control liposome made entirely of PC. The data in panel E demonstrate stepwise increases in daptomycin binding to PG. (F) Structures of our lipid of interest. Statistical analysis was performed with 1-way ANOVA, and the data in every column represent comparisons of the means (*, *P* ≤ 0.05; **, *P* ≤ 0.01; ***, *P* ≤ 0.001; ****, *P* ≤ 0.0001). All tests were performed in buffer containing 100 mM KCl, 10 mM HEPES (pH 7.0), and 2 nM Ca^2+^.

In support of our genomic and lipidomic conclusions, we performed a structure-function analysis of PG, which we show is the target of daptomycin and is necessary and sufficient for daptomycin activity *in vitro*. By combining surface plasmon resonance (SPR) analysis, which measures binding, and the carboxyfluorescein liposome stability assay (CFLSA), which measures activity, we are able to understand the structural interactions of daptomycin with PG. We used 200-nm artificial liposomes of relevant membrane lipid compositions to determine these relationships. Four lipid species—PG, CL, phosphatidic acid (PA), and phosphatidylcholine (PC) (Fig. 7F; see also Fig. S3)—were tested for daptomycin binding affinity. Both PI and Glua-DAG were found in the WT and HLDR isolates, and we did not test them in the next set of experiments because our lipidomics analysis indicated they are not the *in vivo* targets of daptomycin. Three types of liposomes of defined composition were assembled, comprised of PG, CL, and PA combined at a 1:1 molar ratio with PC, and were compared with homogeneous PC-only liposomes. Daptomycin showed a significantly higher affinity to PG liposomes (*P* ≤ 0.0001 and *P* ≤ 0.001) than any of the other lipids tested (Fig. 7). PG has been hypothesized to be an *in vitro* target of daptomycin due to its charged phosphate; however, the lack of binding to PC and the minimal binding to PA (Fig. 7E) indicate that the negatively charged phosphate plays a subordinate role in daptomycin binding. Additionally, when the fatty acyl groups were restricted to the bilayer surface plane, as they are in CL, daptomycin bound with much lower affinity (Fig. 7A to E). When the phosphatidyl-sn-glycerol-3-phosphate glycerol head group was accessible, as it is with PG, daptomycin bound more efficiently (Fig. 7A to E). Also, the daptomycin binding to the 1:1 PG liposomes appeared to saturate with daptomycin above 20 µg/ml, indicating that PG was acting as a binding site for the daptomycin (Fig. 7E). Accordingly, we would expect daptomycin activity to correlate with the binding of PG, CL, PA, and PC, and we tested this through a carboxyfluorescein liposome stability assay (CLFSA).

### CFLSA results indicate that PG is necessary and sufficient for daptomycin activity.

**FIG 8:**
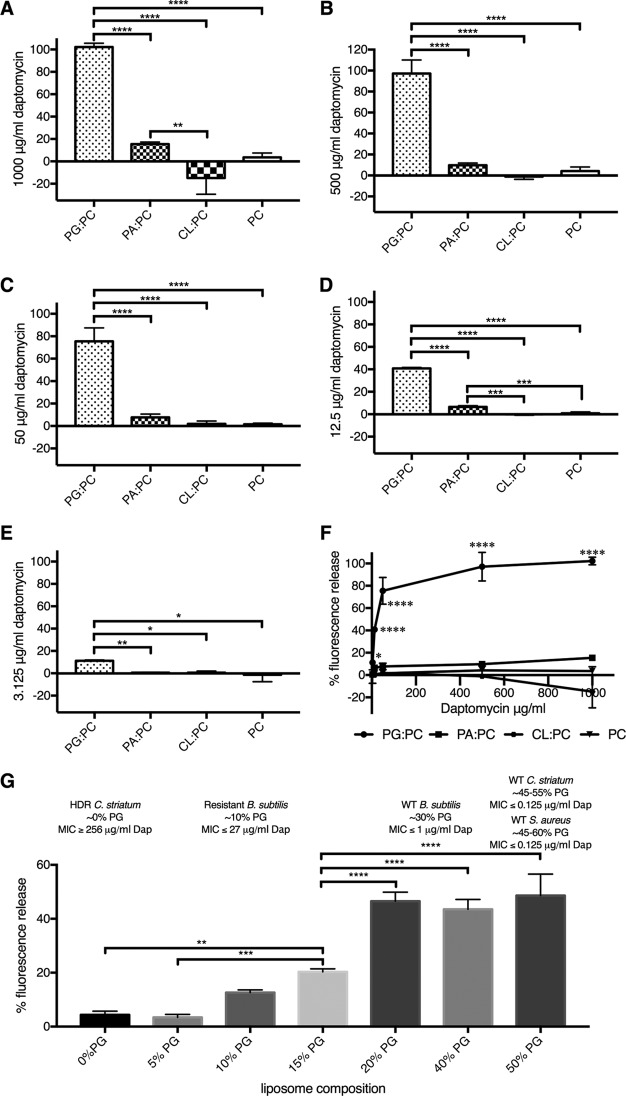
Daptomycin activity across concentrations and liposome content. (A to F) The *y* axis plots percent activity of various concentrations of daptomycin based on absolute fluorescence that is normalized to the fluorescence achieved by the addition of Triton X-100. In panels A to E, the *x* axis shows the types of liposomes tested, which include 1:1 equimolar ratios of PG, PA, or CL to PC and a control liposome made entirely of PC. (F) The *x* axis indicates the concentration of daptomycin added to the different liposome compositions. (G) Relation of percent activity of 35 µg/ml of daptomycin (Dap) based on absolute fluorescence that is normalized to the fluorescence achieved by the addition of Triton X-100 (*y* axis) to the percentage of PG content of the liposome tested with PC contributing the remainder of the required lipid to reach 100% composition (*x* axis). Daptomycin activity against liposomes is correlated with MIC values for WT and daptomycin-resistant bacterial isolates where the percentage of PG content is known. HDR, highly daptomycin resistant. Statistical analysis was performed with 1-way ANOVA, and the data in every column represent comparisons of the means (*, *P* ≤ 0.05; **, *P* ≤ 0.01; ***, *P* ≤ 0.001; ****, *P* ≤ 0.0001). All tests were performed in buffer containing 100 mM KCl, 10 mM HEPES (pH 7.0), and 2 nM Ca2+.

We found that the presence of PG in the bacterial membrane correlates with daptomycin’s bactericidal activity. Carboxyfluorescein liposome stability assays (CLFSA) confirmed PG’s role in daptomycin activity *in vitro*. Liposomes were generated as described above in the presence of self-quenching carboxyfluorescein. Daptomycin was added and interacted with the liposome membrane, releasing and diluting the carboxyfluorescein, which was then unquenched in the buffer, producing dramatically increased fluorescence (53). Daptomycin had higher activity against PG-containing membranes in all cases (Fig. 8A to F) and acted in a concentration-dependent manner against both PG and PA (Fig. 8F). Even though PA had lower binding affinity to daptomycin than CL (Fig. 7E), daptomycin was more active against PA than it was against liposomes containing CL, where there were no available glycerol-3-phosphates extending from the membrane surface for daptomycin binding (Fig. 8A and D). This is consistent with the observed PG-to-CL daptomycin activity relationship. Furthermore, this suggests to us that the larger (4-alkane-chain) CL suppressed daptomycin’s integration with the membrane structure, which is necessary for increased permeability in the CFLSA inhibiting daptomycin activity. We observed a reduction in daptomycin activity in liposomes that contain CL even though they have a higher binding affinity than those containing PA and PC (Fig. 7E and 8A to F). These findings indicate that the conversion of PG to CL can reduce the activity of daptomycin in membranes, providing low-level resistance against daptomycin *in vivo*.

### CFLSA data indicate that the PG concentration predicts daptomycin activity *in vivo.*

We found that daptomycin bactericidal activity is correlated with the percentage of composition of PG in the membrane. Liposomes with 0% to 50% PG were generated as described above, and daptomycin’s activity against those liposomes was tested at a consistent 35 µg/ml daptomycin. At or above 20% PG composition, daptomycin did not show a significant change in activity (Fig. 8G). This observation directly maps to daptomycin’s *in vivo* bactericidal activity as measured by MIC across a number of bacterial species. B. subtilis, C. striatum, and S. aureus strains with 30% to 50% membrane PG (29, 33, 54) content have daptomycin MICs of ≤1 µg/ml. Below 20% PG content, daptomycin’s activity drops precipitously, both *in vitro and in vivo*. Daptomycin-resistant B. subtilis with a PG content of 10% showed a 27-fold increase in MIC (29). This was recapitulated with our artificial liposomes, where equivalent daptomycin reduction resulted in a 3.66-fold decrease in carboxyfluorescein-based fluorescence (Fig. 8G), indicative of a loss in daptomycin activity. When membrane PG composition is ≤5%, daptomycin’s activity is indistinguishable from that seen with a complete absence of PG in the artificial liposome. In HLDR C. striatum, which has 0% PG in its membrane, we saw a ≥4,000 increase in MIC (14, 33) and our data show a 13.5-fold decrease in carboxyfluorescein fluorescence when the liposomes had <5% PG. Thus, the percentage of composition of PG relative to other membrane lipids is predictive of daptomycin susceptibility and activity both *in vivo* and *in vitro*. It also suggests that the CFLSA *in vitro* model is an effective method of studying daptomycin’s interactions with membranes.

## DISCUSSION

C. striatum is an emerging, commensal opportunistic pathogen that has the potential to cause widespread harm in our hospital systems. Rapid adaptive evolution of loss-of-function *pgsA2* (PG synthase) mutations which result in significant loss or removal of membrane PG are necessary and sufficient for high-level daptomycin resistance in C. striatum, which leads to catastrophic daptomycin treatment failure in patients. No additional genomic or transcriptomic compensation mechanisms are evident for the evolved HLDR phenotype. The HLDR mutants also have no changes in cell wall thickness, cell surface charge, conversion of PG to cardiolipin, or membrane shape, which are all mechanisms previously implicated in lower-level daptomycin resistance (19, 20, 22-25, 27, 28, 51, 55-58). Rebalancing of membrane composition to include more PI in the absence of PG as observed by lipidomic profiling likely results in membrane stability. The ability of C. striatum to completely remove PG from its membrane with simple loss-of-function point mutations in PG synthase further demonstrates that PG is the *in vivo* and *in vitro* target of daptomycin. The remarkable ability of C. striatum to remove a membrane phospholipid that had previously been presumed to be necessary could make C. striatum an ideal model for developing new Gram-positive antibiotics such as daptomycin and for studying the potential for resistance to develop.

## MATERIALS AND METHODS

All C. striatum isolates were cultured in BBL Mueller-Hinton II broth (cation-adjusted) (BD) supplemented with calcium to maintain 50 µg/ml calcium.

### Whole-genome sequencing and comparison.

We sequenced 8 sets of C. striatum strains before and after the emergence of high-level resistance to daptomycin. Isolates were sequenced using an Illumina Hi Seq 2500 platform, generating 101-bp paired-end reads. One case of resistance emergence occurred in a patient bloodstream, while the remaining strains evolved resistance during *in vitro* selection. We used the original susceptible patient isolate as our reference strain and assembled the genome *de novo* using SPADES (59). This reference genome was annotated using Prokka v1.12 and the Pfam database (60). We assembled the remaining 15 genomes by mapping to this reference using bowtie2 (61). We identified single nucleotide polymorphisms (SNPs) between the resistant strains and their respective susceptible controls using Pilon (62). The effects of these mutations were predicted using SNPeff (42). All of the genes annotated in the reference genome were clustered by predicted metabolic function using Blast2Go (63). Genes with a predicted loss-of-function mutation were annotated on this metabolic map. *pgsA2* was the only gene with a biosynthetic function predicted to contain loss-of-function mutations in more than one case of HLDR (in fact, *pgsA2* loss-of-function mutations were found in all cases of resistance). A total of 33 other nonsynonymous SNPs were detected across all 8 strain pairs. Only 8 of these SNPs were in genes predicted to affect biosynthetic processes. After clustering by Gene Ontology (GO) was performed, *pgsa2* altering phospholipid biosynthesis was the only metabolic alteration predicted in more than one case of resistance. Phyre2 homology modeling (44) supported predictions that the *pgsA2* mutation in every resistant strain was associated with loss of function.

### Transcriptomic methods.

We performed transcriptomic profiling of the susceptible isolate and the *in vivo* evolved resistant isolate from the original patient in triplicate. Frozen culture was streaked onto plates containing CAMHB plus blood and was grown overnight for single-colony selection and then inoculated into 50 ml CAMHB and grown overnight. The following day, the cultures were diluted to a 0.5 McFarland standard and split into three 100-ml cultures per condition. The diluted cultures were incubated at 37°C with shaking for 1 h. The cells were collected by centrifugation at 200 × *g* for 15 min. The pellets were resuspended in RNAlater (76104 Qiagen) and frozen at −80 until analysis.

We used bead beating and SDS treatment to disrupt the sample cells, phenol:chloroform extraction to remove proteins, and alcohol precipitation followed by DNase treatment to isolate RNA from the frozen samples. rRNA was removed by the use of a Ribo-Zero rRNA removal kit (Epicentre). cDNA libraries were generated from the isolated RNA and amplified as described by Yoneda et al. (46). The double-stranded cDNA libraries were sequenced using a Nextera platform (64) to generate at least 7 million 75-bp reads from each sample.

Reads from the triplicate susceptible and HLDR samples were aligned to the reference genome constructed from the index susceptible isolate using cufflinks (65). Differences in expression between susceptible and HLDR strains were calculated using cuffdiff (66). Fold change was calculated as the resistant-isolate expression level divided by the susceptible-isolate expression level. Expression levels of control housekeeping genes *rpoA* and *gyrA* remained constant in resistant versus susceptible samples (fold changes of 1.02 and 1.00). The statistical significance of changes was calculated using the beta negative binomial distribution previously described by Trapnell et al. (66) and a significance level *P* value of <0.05.

### Zeta potential measurement methods.

We performed surface charge measurement of the susceptible and HLDR strains using zeta potential (67). Frozen culture was streaked onto plates containing CAMHB plus blood and was grown overnight for single-colony selection and then inoculated into 50 ml CAMHB and grown overnight. The following day, the cultures were diluted to a 0.5 McFarland standard and one 4-ml culture was grown per condition. The diluted cultures were incubated at 37°C with shaking for 1 h. A 1-ml volume of the culture was placed in a Malvern zeta-sizing cuvette. Zeta potential (surface charge) was measured using a Zetasizer Nano ZS (ZEN3600) dynamic light-scattering system (Malvern Instruments), and the data were compared between susceptible and HLDR paired strains. The expression levels of genes related to lipid biosynthesis are presented in Fig. 2 (see also Fig. S3 in the supplemental material). Additionally, the genes with the greatest fold changes were determined (Data Set S1).

### Spent-medium growth curve.

C. striatum daptomycin-resistant isolates RP1B, RE4, and RE5 and wild-type daptomycin-susceptible S. aureus ATCC strain 25923 were grown in the presence of cation-adjusted MHB (BBL Mueller-Hinton II broth) with supplemental calcium added to reach 50 mg/liter in the presence of 5 g/ml daptomycin and without daptomycin for 24 h at 37°C with agitation in 10-ml tubes with 5 ml of culture. Uninoculated medium was also incubated with agitation and with and without daptomycin. The spent medium was filtered using a 0.22-μm-pore-size filter and divided into aliquots, placed in a 96-well plate, and seeded with daptomycin-susceptible S. aureus 29213 in triplicate followed by growth at 37°C with agitation. The plate was read every 30 min over 48 h.

### Lipidomic methods.

We performed comparative lipidomics across all four mutation types. Matched WT and HLDR isolates were grown overnight and diluted to an optical density (OD) of 1 in 2.5 ml liquid culture in quintuplicate. Liquid cultures were spun down to form a cell pellet, and whole-cell lipids were extracted using the Bligh-Dyer method (68). Samples were then stored at −20°C until the lipids could be analyzed via liquid chromatography/mass spectrometry (LC/MS). The peak intensities were normalized to 100 for each lipid, with the WT or HLDR isolate being the source of the normalizing lipid. The WT lipids were chosen as the normalizing lipids for PG and cardiolipin because those lipids were most abundant in the WT isolate compared to the HLDR isolate. The HLDR lipids were chosen as the normalizing lipids for CDP-DAG, Glua-DAG, and PI because they were most abundant in the HLDR isolate compared to the WT isolate. Statistical analysis was performed with 1-way analysis of variance (ANOVA), and the data in every column in the figures in which the results of the analysis appear represent comparisons of the means (*, *P* ≤ 0.05; **, *P* ≤ 0.01; ***, *P* ≤ 0.001; ****, *P* ≤ 0.0001).

### Carboxyfluorescein liposome stability assay.

We performed a liposome disruption assay (69) in triplicate to assess the activity of daptomycin with various compositions of liposomes. Equimolar ratios of PG:PC, CL:PC, and PA:PC with a PC-only liposome control were created using the reverse-phase method in the presence of elution buffer containing 100 mM KCl, 10 mM HEPES (pH 7.0), 2 nM Ca^2+^, and carboxyfluorescein (70). Liposomes were then suspended in a buffer solution containing 100 mM KCl, 10 mM HEPES (pH 7.0), and 2 nM Ca^2+^ and subjected to various concentrations of daptomycin from 3.125 µg/ml to 1,000 μg/ml. Levels of fluorescence increases due to daptomycin as a result of carboxyfluorescein release were measured using a Varian Eclipse spectrophotometer with an excitation wavelength of 492 nm and an emission wavelength of 512 nm. Daptomycin activity was measured as a function of normalization to 100% release by Triton X-100. Additionally, to assess percent PG with respect to daptomycin activity, PG:PC liposomes were created in triplicate with various mole fraction ratios converted to various proportions of PG (50%, 40%, 20%, 15%, 10%, 5%, and 0%) and subjected to 35 µg/ml daptomycin. Statistical analysis was performed with 1-way ANOVA, and the data in every column in the figures in which the results of the analysis appear represent comparisons of the means (*, *P* ≤ 0.05; **, *P* ≤ 0.01; ***, *P* ≤ 0.001; ****, *P* ≤ 0.0001).

### Surface plasmon resonance.

We performed surface plasmon resonance analyses (71) in triplicate to assess the binding of daptomycin with various compositions of liposomes. Equimolar ratios (1:1) of PG:PC, CL:PC, and PA:PC with a PC-only liposome control were created using the reverse-phase method (70). Liposomes were bound to a carboxymethyl dextran hydrogel surface sensor chip that was treated with sphingosine and subjected to various concentrations of daptomycin (3.125 µg/ml to 35 μg/ml) diluted in buffer containing 100 mM KCl, 10 mM HEPES (pH 7.0), and 2 nM Ca^2+^. Baseline, stable liposome, and peak daptomycin binding readings were collected. Daptomycin binding was normalized to liposome binding, and the data are presented as the level of daptomycin/lipid unit. Statistical analysis was performed with 1-way ANOVA, and the data in every column in the figures in which the results of the analysis appear represent comparisons of the means (*, *P* ≤ 0.05; **, *P* ≤ 0.01; ***, *P* ≤ 0.001; ****, *P* ≤ 0.0001).

### Accession number(s).

All nucleotide sequences generated during this study have been uploaded to NCBI under BioProject accession no. PRJNA420593.

## References

[1] O’Neill J 2015 Antimicrobials in agriculture and environment: reducing unnecessary use and waste. The review on antimicrobial resistance. https://amr-review.org/sites/default/files/Antimicrobials%20in%20agriculture%20and%20the%20environment%20-%20Reducing%20unnecessary%20use%20and%20waste.pdf.

[2] O’Neill J 2014 Antimicrobial resistance: tackling a crisis for the health and wealth of nations. The review on antimicrobial resistance. https://amr-review.org/sites/default/files/AMR%20Review%20Paper%20-%20Tackling%20a%20crisis%20for%20the%20health%20and%20wealth%20of%20nations_1.pdf.

[3] Maragakis LL, Perencevich EN, Cosgrove SE 2008 Clinical and economic burden of antimicrobial resistance. Expert Rev Anti Infect Ther 6:751–763. doi:10.1586/14787210.6.5.751. 18847410

[4] Gelband H, Miller-Petrie M, Pant S, Gandra S, Levinson J, Barter D, White A, Laxminarayan R 2015 State of the world’s antibiotics, 2015. The Center for Disease Dynamics, Economics & Policy, Washington, DC.

[5] Srinivasan A 2017 Antibiotic stewardship: why we must, how we can. Cleve Clin J Med 84:673–679. doi:10.3949/ccjm.84gr.17003. 28885907 PMC6492617

[6] Srinivasan A, Davidson LE 2017 Improving patient safety through antibiotic stewardship: the Veterans Health Administration leads the way, again. Infect Control Hosp Epidemiol 38:521–523. doi:10.1017/ice.2017.38. 28421978 PMC11320708

[7] Cotroneo N, Harris R, Perlmutter N, Beveridge T, Silverman JA 2008 Daptomycin exerts bactericidal activity without lysis of Staphylococcus aureus. Antimicrob Agents Chemother 52:2223–2225. doi:10.1128/AAC.01410-07. 18378708 PMC2415783

[8] Mascio CT, Alder JD, Silverman JA 2007 Bactericidal action of daptomycin against stationary-phase and nondividing Staphylococcus aureus cells. Antimicrob Agents Chemother 51:4255–4260. doi:10.1128/AAC.00824-07. 17923487 PMC2167999

[9] Jorgensen JH, Maher LA, Redding JS 1987 In vitro activity of LY146032 (daptomycin) against selected aerobic bacteria. Eur J Clin Microbiol 6:91–96. doi:10.1007/BF02097209. 3032612

[10] Jorgensen JH, Crawford SA, Kelly CC, Patterson JE 2003 In vitro activity of daptomycin against vancomycin-resistant enterococci of various Van types and comparison of susceptibility testing methods. Antimicrob Agents Chemother 47:3760–3763. doi:10.1128/AAC.47.12.3760-3763.2003. 14638478 PMC296189

[11] Humphries RM, Kelesidis T, Tewhey R, Rose WE, Schork N, Nizet V, Sakoulas G 2012 Genotypic and phenotypic evaluation of the evolution of high-level daptomycin nonsusceptibility in vancomycin-resistant Enterococcus faecium. Antimicrob Agents Chemother 56:6051–6053. doi:10.1128/AAC.01318-12. 22948885 PMC3486580

[12] Ho SW, Jung D, Calhoun JR, Lear JD, Okon M, Scott WR, Hancock RE, Straus SK 2008 Effect of divalent cations on the structure of the antibiotic daptomycin. Eur Biophys J 37:421–433. doi:10.1007/s00249-007-0227-2. 17968536

[13] Hobbs JK, Miller K, O’Neill AJ, Chopra I 2008 Consequences of daptomycin-mediated membrane damage in Staphylococcus aureus. J Antimicrob Chemother 62:1003–1008. doi:10.1093/jac/dkn321. 18669516

[14] McElvania TeKippe E, Thomas BS, Ewald GA, Lawrence SJ, Burnham CA 2014 Rapid emergence of daptomycin resistance in clinical isolates of Corynebacterium striatum … a cautionary tale. Eur J Clin Microbiol Infect Dis 33:2199–2205. doi:10.1007/s10096-014-2188-6. 24973133 PMC4331070

[15] Akins RL, Katz BD, Monahan C, Alexander D 2015 Characterization of high-level daptomycin resistance in viridans group streptococci developed upon in vitro exposure to daptomycin. Antimicrob Agents Chemother 59:2102–2112. doi:10.1128/AAC.04219-14. 25624330 PMC4356810

[16] García-de-la-Mària C, Pericas JM, Del Río A, Castañeda X, Vila-Farrés X, Armero Y, Espinal PA, Cervera C, Soy D, Falces C, Ninot S, Almela M, Mestres CA, Gatell JM, Vila J, Moreno A, Marco F, Miró JM; Hospital Clinic Experimental Endocarditis Study Group 2013 Early in vitro and in vivo development of high-level daptomycin resistance is common in mitis group streptococci after exposure to daptomycin. Antimicrob Agents Chemother 57:2319–2325. doi:10.1128/AAC.01921-12. 23478959 PMC3632914

[17] OptumRx 2016 Cubicin (daptomycin)—first-time generic. OptumRx Clinical Services Department. https://professionals.optumrx.com/content/dam/optum3/professional-optumrx/news/rxnews/new-generics/newgenerics_cubicin_2016-0915.pdf. Accessed 1 November 2016.

[18] Sauermann R, Rothenburger M, Graninger W, Joukhadar C 2008 Daptomycin: a review 4 years after first approval. Pharmacology 81:79–91. doi:10.1159/000109868. 17940348

[19] Gaupp R, Lei S, Reed JM, Peisker H, Boyle-Vavra S, Bayer AS, Bischoff M, Herrmann M, Daum RS, Powers R, Somerville GA 2015 Staphylococcus aureus metabolic adaptations during the transition from a daptomycin susceptibility phenotype to a daptomycin nonsusceptibility phenotype. Antimicrob Agents Chemother 59:4226–4238. doi:10.1128/AAC.00160-15. 25963986 PMC4468685

[20] Reyes J, Panesso D, Tran TT, Mishra NN, Cruz MR, Munita JM, Singh KV, Yeaman MR, Murray BE, Shamoo Y, Garsin D, Bayer AS, Arias CA 2015 A liaR deletion restores susceptibility to daptomycin and antimicrobial peptides in multidrug-resistant Enterococcus faecalis. J Infect Dis 211:1317–1325. doi:10.1093/infdis/jiu602. 25362197 PMC4402337

[21] Mishra NN, Bayer AS, Weidenmaier C, Grau T, Wanner S, Stefani S, Cafiso V, Bertuccio T, Yeaman MR, Nast CC, Yang SJ 2014 Phenotypic and genotypic characterization of daptomycin-resistant methicillin-resistant Staphylococcus aureus strains: relative roles of mprF and dlt operons. PLoS One 9:e107426. doi:10.1371/journal.pone.0107426. 25226591 PMC4166420

[22] Raad I, Hanna H, Jiang Y, Dvorak T, Reitzel R, Chaiban G, Sherertz R, Hachem R 2007 Comparative activities of daptomycin, linezolid, and tigecycline against catheter-related methicillin-resistant Staphylococcus bacteremic isolates embedded in biofilm. Antimicrob Agents Chemother 51:1656–1660. doi:10.1128/AAC.00350-06. 17353249 PMC1855569

[23] Quinn B, Hussain S, Malik M, Drlica K, Zhao X 2007 Daptomycin inoculum effects and mutant prevention concentration with Staphylococcus aureus. J Antimicrob Chemother 60:1380–1383. doi:10.1093/jac/dkm375. 17905797

[24] Bayer AS, Schneider T, Sahl HG 2013 Mechanisms of daptomycin resistance in Staphylococcus aureus: role of the cell membrane and cell wall. Ann N Y Acad Sci 1277:139–158. doi:10.1111/j.1749-6632.2012.06819.x. 23215859 PMC3556211

[25] Mishra NN, Yang SJ, Sawa A, Rubio A, Nast CC, Yeaman MR, Bayer AS 2009 Analysis of cell membrane characteristics of in vitro-selected daptomycin-resistant strains of methicillin-resistant Staphylococcus aureus. Antimicrob Agents Chemother 53:2312–2318. doi:10.1128/AAC.01682-08. 19332678 PMC2687258

[26] Li S, Yin Y, Chen H, Wang Q, Wang X, Wang H 2017 Fitness cost of daptomycin-resistant Staphylococcus aureus obtained from in vitro daptomycin selection pressure. Front Microbiol 8:2199. doi:10.3389/fmicb.2017.02199. 29170657 PMC5684181

[27] D’Costa VM, Mukhtar TA, Patel T, Koteva K, Waglechner N, Hughes DW, Wright GD, De Pascale G 2012 Inactivation of the lipopeptide antibiotic daptomycin by hydrolytic mechanisms. Antimicrob Agents Chemother 56:757–764. doi:10.1128/AAC.05441-11. 22083474 PMC3264212

[28] D’Costa VM, McGrann KM, Hughes DW, Wright GD 2006 Sampling the antibiotic resistome. Science 311:374–377. doi:10.1126/science.1120800. 16424339

[29] Hachmann AB, Sevim E, Gaballa A, Popham DL, Antelmann H, Helmann JD 2011 Reduction in membrane phosphatidylglycerol content leads to daptomycin resistance in Bacillus subtilis. Antimicrob Agents Chemother 55:4326–4337. doi:10.1128/AAC.01819-10. 21709092 PMC3165287

[30] Hachmann AB, Angert ER, Helmann JD 2009 Genetic analysis of factors affecting susceptibility of Bacillus subtilis to daptomycin. Antimicrob Agents Chemother 53:1598–1609. doi:10.1128/AAC.01329-08. 19164152 PMC2663116

[31] Hines KM, Waalkes A, Penewit K, Holmes EA, Salipante SJ, Werth BJ, Xu L 2017 Characterization of the mechanisms of daptomycin resistance among Gram-positive bacterial pathogens by multidimensional lipidomics. mSphere 2:e00492-17. doi:10.1128/mSphere.00492-17. 29242835 PMC5729219

[32] Tran TT, Jaijakul S, Lewis CT, Diaz L, Panesso D, Kaplan HB, Murray BE, Wanger A, Arias CA 2012 Native valve endocarditis caused by Corynebacterium striatum with heterogeneous high-level daptomycin resistance: collateral damage from daptomycin therapy? Antimicrob Agents Chemother 56:3461–3464. doi:10.1128/AAC.00046-12. 22450978 PMC3370765

[33] McMullen AR, Anderson N, Wallace MA, Shupe A, Burnham CA 2017 When good bugs go bad: epidemiology and antimicrobial resistance profiles of Corynebacterium striatum, an emerging multidrug-resistant, opportunistic pathogen. Antimicrob Agents Chemother 61:e01111-17. doi:10.1128/AAC.01111-17. 28848008 PMC5655097

[34] Oh J, Byrd AL, Deming C, Conlan S; NISC Comparative Sequencing Program, Kong HH, Segre JA 2014 Biogeography and individuality shape function in the human skin metagenome. Nature 514:59–64. doi:10.1038/nature13786. 25279917 PMC4185404

[35] Wong KY, Chan YC, Wong CY 2010 Corynebacterium striatum as an emerging pathogen. J Hosp Infect 76:371–372. doi:10.1016/j.jhin.2010.05.018. 20688419

[36] Gomila M, Renom F, Gallegos MDC, Garau M, Guerrero D, Soriano JB, Lalucat J 2012 Identification and diversity of multiresistant Corynebacterium striatum clinical isolates by MALDI-TOF mass spectrometry and by a multigene sequencing approach. BMC Microbiol 12:52. doi:10.1186/1471-2180-12-52. 22475029 PMC3348057

[37] Mattos-Guaraldi AL, Guimarães LC, Santos CS, Veras AA, Carneiro AR, Soares SC, Ramos JN, Souza C, Vieira VV, Hirata R Jr, Azevedo V, Pacheco LG, Silva A, Ramos RT 2015 Draft genome sequence of Corynebacterium striatum 1961 BR-RJ/09, a multidrug-susceptible strain isolated from the urine of a hospitalized 37-year-old female patient. Genome Announc 3:e00869-15. doi:10.1128/genomeA.00869-15. 26251495 PMC4541279

[38] de Souza C, Faria YV, Sant’Anna LDO, Viana VG, Seabra SH, Souza MC, Vieira VV, Hirata Júnior R, Moreira LDO, Mattos-Guaraldi AL 2015 Biofilm production by multiresistant Corynebacterium striatum associated with nosocomial outbreak. Mem Inst Oswaldo Cruz 110:242–248. doi:10.1590/0074-02760140373. 25946249 PMC4489456

[39] Wang J, Wang Y, Du X, Cui J, Wang K, Zhang L, Han Y 2016 Rapid transmission of multidrug-resistant Corynebacterium striatum among susceptible patients in a tertiary hospital in China. J Infect Dev Ctries 10:1299–1305. doi:10.3855/jidc.7577. 28036309

[40] Werth BJ, Hahn WO, Butler-Wu SM, Rakita RM 2016 Emergence of high-level daptomycin resistance in Corynebacterium striatum in two patients with left ventricular assist device infections. Microb Drug Resist 22:233–237. doi:10.1089/mdr.2015.0208. 26544621 PMC4834517

[41] Hahn WO, Werth BJ, Butler-Wu SM, Rakita RM 2016 Multidrug-resistant Corynebacterium striatum associated with increased use of parenteral antimicrobial drugs. Emerg Infect Dis 22. doi:10.3201/eid2211.160141. PMC5088002 27767926

[42] Cingolani P, Platts A, Wang LL, Coon M, Nguyen T, Wang L, Land SJ, Lu X, Ruden DM 2012 A program for annotating and predicting the effects of single nucleotide polymorphisms, SnpEff: SNPs in the genome of Drosophila melanogaster strain w1118; iso-2; iso-3. Fly 6:80–92. doi:10.4161/fly.19695. 22728672 PMC3679285

[43] Cingolani P, Patel VM, Coon M, Nguyen T, Land SJ, Ruden DM, Lu X 2012 Using Drosophila melanogaster as a model for genotoxic chemical mutational studies with a new program, SnpSift. Front Genet 3:35. doi:10.3389/fgene.2012.00035. 22435069 PMC3304048

[44] Kelley LA, Mezulis S, Yates CM, Wass MN, Sternberg MJ 2015 The Phyre2 web portal for protein modeling, prediction and analysis. Nat Protoc 10:845–858. doi:10.1038/nprot.2015.053. 25950237 PMC5298202

[45] Kelley LA, Sternberg MJ 2009 Protein structure prediction on the Web: a case study using the Phyre server. Nat Protoc 4:363–371. doi:10.1038/nprot.2009.2. 19247286

[46] Yoneda A, Henson WR, Goldner NK, Park KJ, Forsberg KJ, Kim SJ, Pesesky MW, Foston M, Dantas G, Moon TS 2016 Comparative transcriptomics elucidates adaptive phenol tolerance and utilization in lipid-accumulating Rhodococcus opacus PD630. Nucleic Acids Res 44:2240–2254. doi:10.1093/nar/gkw055. 26837573 PMC4797299

[47] Mishra NN, Bayer AS 2013 Correlation of cell membrane lipid profiles with daptomycin resistance in methicillin-resistant Staphylococcus aureus. Antimicrob Agents Chemother 57:1082–1085. doi:10.1128/AAC.02182-12. 23254419 PMC3553710

[48] Peschel A, Collins LV 2001 Staphylococcal resistance to antimicrobial peptides of mammalian and bacterial origin. Peptides 22:1651–1659. doi:10.1016/S0196-9781(01)00500-9. 11587793

[49] Staubitz P, Neumann H, Schneider T, Wiedemann I, Peschel A 2004 MprF-mediated biosynthesis of lysylphosphatidylglycerol, an important determinant in staphylococcal defensin resistance. FEMS Microbiol Lett 231:67–71. doi:10.1016/S0378-1097(03)00921-2. 14769468

[50] Ernst CM, Staubitz P, Mishra NN, Yang SJ, Hornig G, Kalbacher H, Bayer AS, Kraus D, Peschel A 2009 The bacterial defensin resistance protein MprF consists of separable domains for lipid lysinylation and antimicrobial peptide repulsion. PLoS Pathog 5:e1000660. doi:10.1371/journal.ppat.1000660. 19915718 PMC2774229

[51] Zhang T, Muraih JK, Tishbi N, Herskowitz J, Victor RL, Silverman J, Uwumarenogie S, Taylor SD, Palmer M, Mintzer E 2014 Cardiolipin prevents membrane translocation and permeabilization by daptomycin. J Biol Chem 289:11584–11591. doi:10.1074/jbc.M114.554444. 24616102 PMC4002069

[52] Rubio A, Moore J, Varoglu M, Conrad M, Chu M, Shaw W, Silverman JA 2012 LC-MS/MS characterization of phospholipid content in daptomycin-susceptible and -resistant isolates of Staphylococcus aureus with mutations in mprF. Mol Membr Biol 29:1–8. doi:10.3109/09687688.2011.640948. 22276671

[53] Saito M, Korsmeyer SJ, Schlesinger PH 2000 BAX-dependent transport of cytochrome c reconstituted in pure liposomes. Nat Cell Biol 2:553–555. doi:10.1038/35019596. 10934477

[54] Randall CP, Mariner KR, Chopra I, O’Neill AJ 2013 The target of daptomycin is absent from Escherichia coli and other gram-negative pathogens. Antimicrob Agents Chemother 57:637–639. doi:10.1128/AAC.02005-12. 23114759 PMC3535891

[55] Mishra B, Lushnikova T, Wang G 2015 Small lipopeptides possess anti-biofilm capability comparable to daptomycin and vancomycin. RSC Adv 5:59758–59769. doi:10.1039/C5RA07896B. 26257894 PMC4524557

[56] Bayer AS, Mishra NN, Sakoulas G, Nonejuie P, Nast CC, Pogliano J, Chen KT, Ellison SN, Yeaman MR, Yang SJ 2014 Heterogeneity of mprF sequences in methicillin-resistant Staphylococcus aureus clinical isolates: role in cross-resistance between daptomycin and host defense antimicrobial peptides. Antimicrob Agents Chemother 58:7462–7467. doi:10.1128/AAC.03422-14. 25288091 PMC4249560

[57] Bayer AS, Mishra NN, Cheung AL, Rubio A, Yang SJ 2016 Dysregulation of mprF and dltABCD expression among daptomycin-non-susceptible MRSA clinical isolates. J Antimicrob Chemother 71:2100–2104. doi:10.1093/jac/dkw142. 27121398 PMC4954926

[58] Bayer AS, Mishra NN, Chen L, Kreiswirth BN, Rubio A, Yang SJ 2015 Frequency and distribution of single-nucleotide polymorphisms within mprF in methicillin-resistant Staphylococcus aureus clinical isolates and their role in cross-resistance to daptomycin and host defense antimicrobial peptides. Antimicrob Agents Chemother 59:4930–4937. doi:10.1128/AAC.00970-15. 26055370 PMC4505294

[59] Bankevich A, Nurk S, Antipov D, Gurevich AA, Dvorkin M, Kulikov AS, Lesin VM, Nikolenko SI, Pham S, Prjibelski AD, Pyshkin AV, Sirotkin AV, Vyahhi N, Tesler G, Alekseyev MA, Pevzner PA 2012 SPAdes: a new genome assembly algorithm and its applications to single-cell sequencing. J Comput Biol 19:455–477. doi:10.1089/cmb.2012.0021. 22506599 PMC3342519

[60] Seemann T 2014 Prokka: rapid prokaryotic genome annotation. Bioinformatics 30:2068–2069. doi:10.1093/bioinformatics/btu153. 24642063

[61] Langmead B, Salzberg SL 2012 Fast gapped-read alignment with Bowtie 2. Nat Methods 9:357–359. doi:10.1038/nmeth.1923. 22388286 PMC3322381

[62] Walker BJ, Abeel T, Shea T, Priest M, Abouelliel A, Sakthikumar S, Cuomo CA, Zeng Q, Wortman J, Young SK, Earl AM 2014 Pilon: an integrated tool for comprehensive microbial variant detection and genome assembly improvement. PLoS One 9:e112963. doi:10.1371/journal.pone.0112963. 25409509 PMC4237348

[63] Conesa A, Götz S, García-Gómez JM, Terol J, Talón M, Robles M 2005 Blast2GO: a universal tool for annotation, visualization and analysis in functional genomics research. Bioinformatics 21:3674–3676. doi:10.1093/bioinformatics/bti610. 16081474

[64] Baym M, Kryazhimskiy S, Lieberman TD, Chung H, Desai MM, Kishony R 2015 Inexpensive multiplexed library preparation for megabase-sized genomes. PLoS One 10:e0128036. doi:10.1371/journal.pone.0128036. 26000737 PMC4441430

[65] Trapnell C, Williams BA, Pertea G, Mortazavi A, Kwan G, van Baren MJ, Salzberg SL, Wold BJ, Pachter L 2010 Transcript assembly and quantification by RNA-Seq reveals unannotated transcripts and isoform switching during cell differentiation. Nat Biotechnol 28:511–515. doi:10.1038/nbt.1621. 20436464 PMC3146043

[66] Trapnell C, Hendrickson DG, Sauvageau M, Goff L, Rinn JL, Pachter L 2013 Differential analysis of gene regulation at transcript resolution with RNA-seq. Nat Biotechnol 31:46–53. doi:10.1038/nbt.2450. 23222703 PMC3869392

[67] Kaisersberger Vincek M, Mor A, Gorgieva S, Kokol V 2017 Antibacterial activity and cytotoxycity of gelatine-conjugated lysine-based peptides. J Biomed Mater Res A 105:3110–3126. doi:10.1002/jbm.a.36164. 28771959

[68] Bligh EG, Dyer WJ 1959 A rapid method of total lipid extraction and purification. Can J Biochem Physiol 37:911–917. doi:10.1139/o59-099. 13671378

[69] Jimah JR, Schlesinger PH, Tolia NH 2017 Liposome disruption assay to examine lytic properties of biomolecules. Bio Protoc 7:e2433. doi:10.21769/BioProtoc.2433. PMC5602583 28932762

[70] Szoka F Jr, Papahadjopoulos D 1978 Procedure for preparation of liposomes with large internal aqueous space and high capture by reverse-phase evaporation. Proc Natl Acad Sci U S A 75:4194–4198. doi:10.1073/pnas.75.9.4194. 279908 PMC336078

[71] Kinouchi H, Onishi M, Kamimori H 2013 Lipid membrane-binding properties of daptomycin using surface plasmon resonance. Anal Sci 29:297–301. doi:10.2116/analsci.29.297. 23474718

[72] Chatzopoulou M, Koufakis T, Voulgaridi I, Gabranis I, Tsiakalou M 2016 A case of fatal sepsis due to multidrug-resistant Corynebacterium striatum. Hippokratia 20:67–69. 27895446 PMC5074401

