# Supplementary figures and images for: Mechanism of High-Level Daptomycin Resistance in Corynebacterium striatum
Source: mSphere. 2018 Aug 8;3(4):e00371-18. doi: 10.1128/mSphereDirect.00371-18 (PMC6083094; doi:10.1128/mSphereDirect.00371-18)

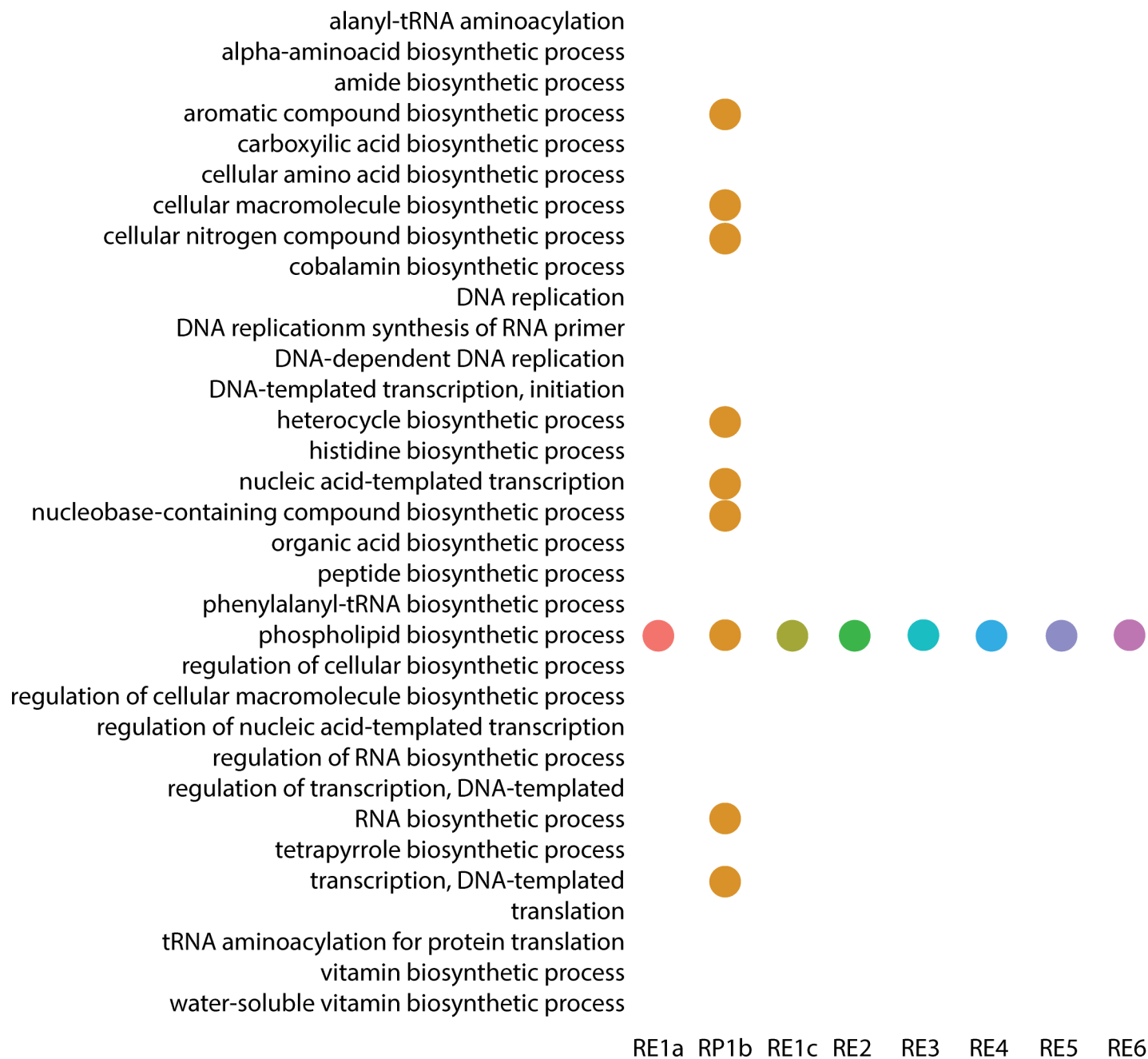

Supplement: FIG S1 [file sph004182609sf1.pdf]
