# Supplementary material for: Mechanism of High-Level Daptomycin Resistance in Corynebacterium striatum
Source: mSphere. 2018 Aug 8;3(4):e00371-18. doi: 10.1128/mSphereDirect.00371-18 (PMC6083094; doi:10.1128/mSphereDirect.00371-18)
Supplement: FIG S2 [file sph004182609sf2.pdf]

A Phosphatidylinositol Lipid Synthesis Pathway

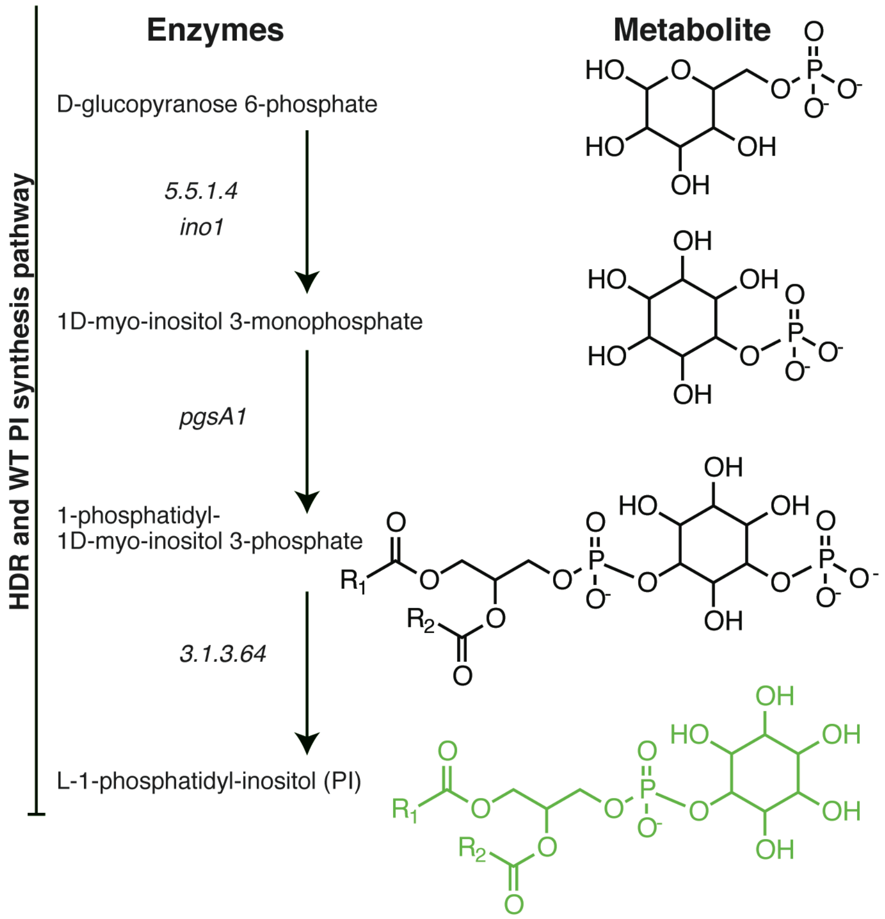

B

Gene Name	Transcript Fold Change Between WP1a and RP1b
pgsA1	1.075
rpoA	1.021
