# Supplementary material for: Mechanism of High-Level Daptomycin Resistance in Corynebacterium striatum
Source: mSphere. 2018 Aug 8;3(4):e00371-18. doi: 10.1128/mSphereDirect.00371-18 (PMC6083094; doi:10.1128/mSphereDirect.00371-18)
Supplement: FIG S3 [file sph004182609sf3.pdf]

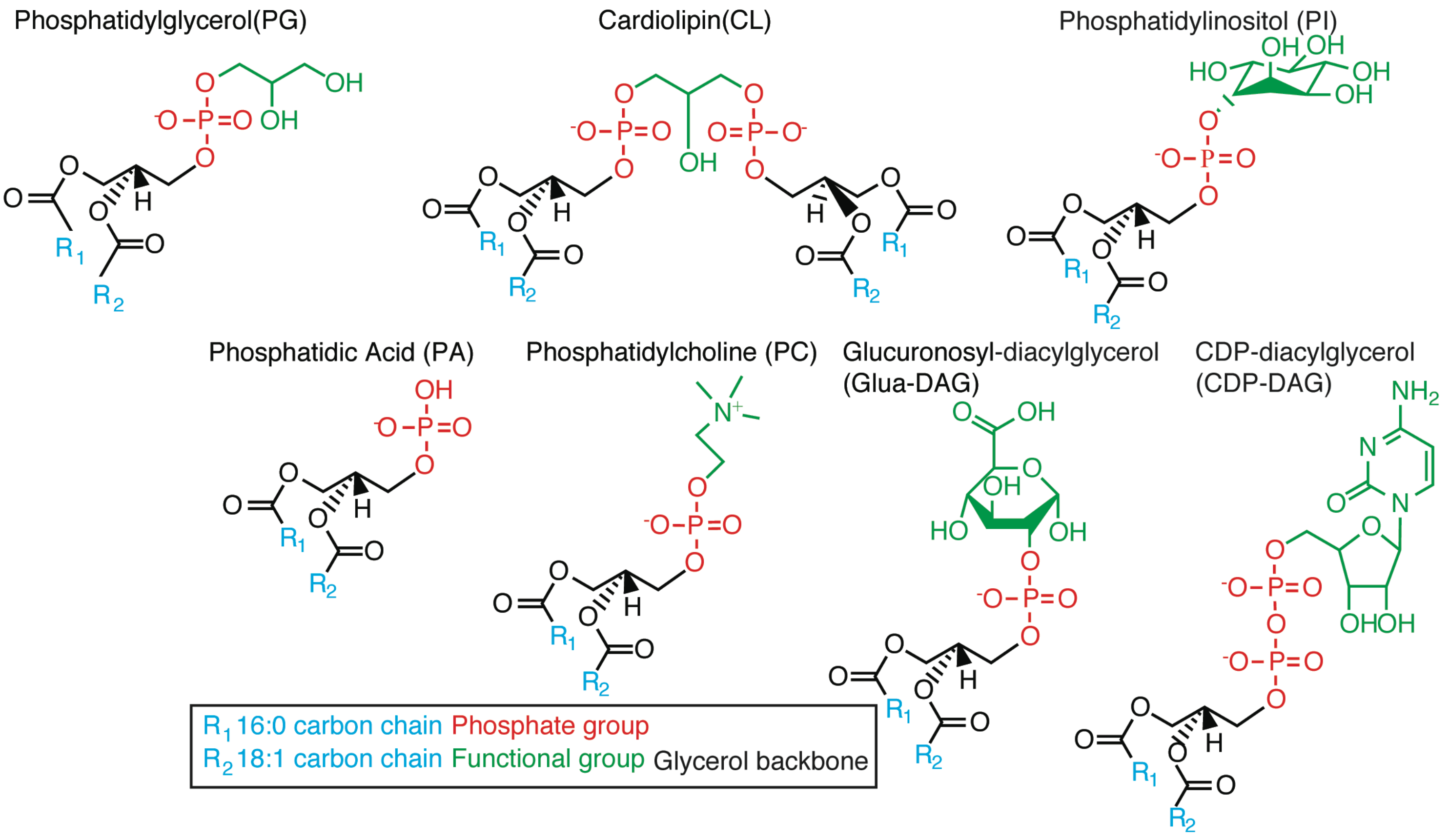
Phosphatidylglycerol(PG)
Cardiolipin(CL)
Phosphatidylinositol (PI)
Phosphatidic Acid (PA)
Phosphatidylcholine (PC)
Glucuronosyl-diacylglycerol (Glua-DAG)
CDP-diacylglycerol (CDP-DAG)
R1 16:0 carbon chain
Phosphate group
R2 18:1 carbon chain
Functional group
Glycerol backbone
